# Cardiac MRI correlates of diastolic left ventricular function assessment by echocardiography

**DOI:** 10.1186/1532-429X-15-S1-E53

**Published:** 2013-01-30

**Authors:** Shahryar G Saba, Sohae Chung, Stephanie Tseng, Sharath Bhagavatula, Robert Donnino, Monvadi B Srichai, Muhamed Saric, Stuart Katz, Leon Axel

**Affiliations:** 1 Medicine, New York University School of Medicine, New York, NY, USA; 2 Radiology, New York University School of Medicine, New York, NY, USA

## Background

Transthoracic echocardiography (TTE) provides non-invasive measures of diastolic left ventricular (LV) function by assessing mitral inflow and mitral annular motion. Given the excellent spatial and temporal resolution of CMR, we developed a novel method to calculate several correlative measures of diastolic and systolic LV function. The maximum velocity of the atrioventricular junction (AVJ) during early diastole represents a correlate of the maximum velocity of the mitral annulus (e'). To determine the correlation between CMR and TTE indices of diastolic function, we performed a retrospective analysis.

## Methods

**Figure 1:**
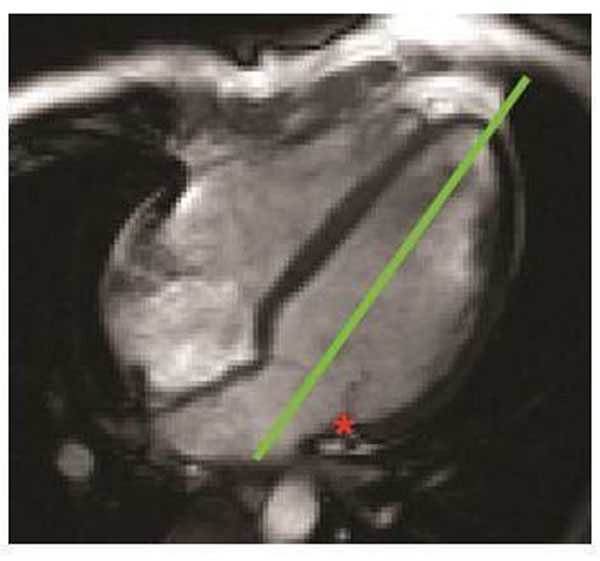
AVJ (red asterisk) motion evaluated on 4-chamber cine CMR view.

**Figure 2:**
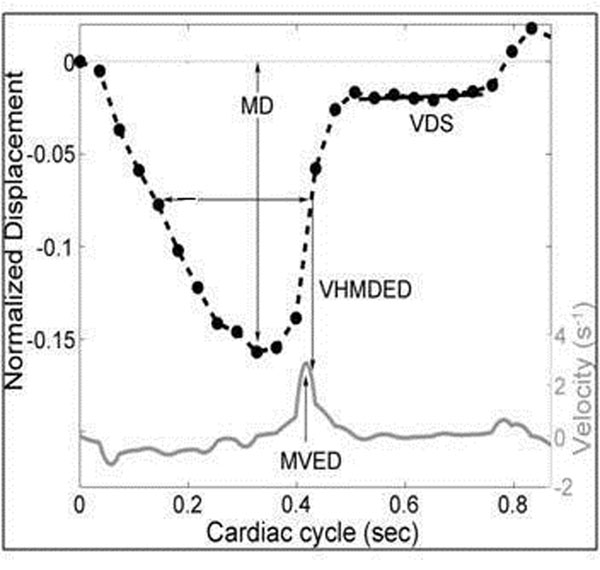
AVJ displacement and derived velocities throughout the cardiac cycle.

We evaluated 50 consecutive patients who underwent TTE and CMR examinations within 30 days. The longitudinal motion of the lateral atrioventricular junction (AVJ) was tracked at 25 times through the cardiac cycle using the 4-chamber cine view. The baseline position of the AVJ was defined at end diastole and its longitudinal displacement was measured relative to a reference line drawn between the LV apex and the midpoint of the mitral annulus (Figure 1). Based on the resulting plot of AVJ position versus time (Figure 2) three motion variables were calculated: maximum longitudinal displacement (MD), maximum velocity during early diastole (MVED), and the slope of the best fit line of displacement in diastasis (VDS). Each TTE was assessed for multiple measures of diastolic function including: mitral inflow deceleration time (DT), peak velocity of mitral inflow during passive filling (E) and with atrial contraction (A), peak velocity of the lateral mitral annulus during passive filling (e') and with atrial contraction (a'). Ejection fractions (EFs) were determined by CMR. The CMR correlates of diastolic left ventricular function were compared to TTE using Pearson's correlation coefficient (r).

## Results

The mean and standard deviation EF determined by CMR was found to be 38% ± 15%. Significant correlations were found between EF and MD (r = 0.604, p < 0.001), both measures of longitudinal LV systolic function, as well as MVED and e' (r = 0.362, p = 0.030), which reflect peak mitral annular velocities during early, passive diastolic filling. A negative correlation was observed between MVED and E/e' (r = -0.425, p = 0.010), suggesting an inverse relationship between peak AVJ velocity during early diastole and left atrial pressure. Significant correlations were also noted for VDS and e' (r = 0.473, p = 0.004), VDS and a' (r = 0.382, p = 0.045), as well as VDS/MVED and DT (r = 0.350, p = 0.034).

## Conclusions

Maximum displacement of the AVJ determined by CMR correlates with EF and serves as a simple measure of LV systolic function. MVED provides a novel CMR correlate of the tissue Doppler echocardiography measure e'. Assessment of AVJ motion variables may provide additional CMR metrics of systolic and diastolic function without increasing scanning time.

## Funding

NIH 1R21HL108218-01.

